# Evidence-Based Medicine: Past, Present, Future

**DOI:** 10.3390/jcm14145094

**Published:** 2025-07-17

**Authors:** Filippos Triposkiadis, Dirk L. Brutsaert

**Affiliations:** 1 Department of Cardiology, Faculty of Medicine, School of Health Sciences, University of Thessaly, Biopolis, 41500 Larissa, Greece; 2 Department of Cardiology, European University Cyprus, 1516 Nicosia, Cyprus; 3 Department of Medicine & Cardiology, University of Antwerp, 2000 Antwerpen, Belgium; dirk.brutsaert@skynet.be

**Keywords:** evidence-based medicine, healthcare, big data

## Abstract

Early medical traditions include those of ancient Babylonia, China, Egypt, and India. The roots of modern Western medicine, however, go back to ancient Greece. During the Renaissance, physicians increasingly relied on observation and experimentation to understand the human body and develop new techniques for diagnosis and treatment. The discovery of antibiotics, antiseptics, and other drugs in the 19th century accelerated the development of modern medicine, the latter being fueled further by advances in technology, research, a better understanding of the human body, and, most recently, the introduction of evidence-based medicine (EBM). The EBM model de-emphasized intuition, unsystematic clinical experience, and pathophysiologic rationale as sufficient grounds for clinical decision-making and stressed the examination of evidence from clinical research. A later EBM model additionally incorporated clinical expertise and the latest model of EBM patients’ preferences and actions. In this review article, we argue that in the era of precision medicine, major EBM principles must be based on (a) the systematic identification, analysis, and utility of big data using artificial intelligence; (b) the magnifying effect of medical interventions by means of the physician–patient interaction, the latter being guided by the physician’s expertise, intuition, and philosophical beliefs; and (c) the patient preferences, since, in healthcare under precision medicine, the patient will be a central stakeholder contributing data and actively participating in shared decision-making.

## 1. Introduction

Early medical traditions include those of ancient Babylonia, China, Egypt, and India. In Babylonia Esangil-kin-apli (1068–1047 B.C.E.), a physician who served the king during the first Babylonian dynasty (around 1069–1046 B.C.E.) compiled the *Diagnostic Handbook*, a medical treatise based on a logical set of axioms and assumptions, including the need to evaluate patient symptomatology to come up with a diagnosis [1].

The foundational text of traditional Chinese medicine, the *Huangdi Neijing* (given the title *The Yellow Emperor’s Classic of Medicine* in one of the latest translations), is an ancient treatise on health and disease based on Taoist philosophy and is credited to the Chinese emperor Huangdi around 2600 B.C.E. [2]. However, Huangdi is a semi-mythical figure, and the book probably dates from around 300 B.C.E. and may be a compilation of the writings of several authors.

Medicine in ancient Egypt had been practiced since the earliest, prehistoric days, many millennia before Christ, and was quite developed in later periods, as evident from the skeletal findings, surgical instruments found in tombs, wall printings, inscription reliefs, and, importantly, medical papyri. The earliest known physician by name in ancient Egypt was Imhotep, who served under the pharaoh Djoser (reigned 2667–2648 B.C.E.) and was credited as being the founder of ancient Egyptian medicine and the author of the *Edwin Smith Papyrus*, which describes the examination, diagnosis, treatment, and prognosis of numerous ailments [3].

One of the first Indian textbooks is the *Atharvaveda* (AV), a sacred text of Hinduism dating from the middle Vedic age (1200–900 B.C.E.). The AV is a curious compendium of medicine in its various stages of evolution and contains the most primitive as well as some of the most highly developed stages of therapy [4].

The roots of modern Western medicine go back to ancient Greece. Hippocrates (460–370 B.C.E.), considered one of the fathers of medicine, claimed that all diseases are due to natural causes. Establishment of diagnosis, prognosis, and treatment should, therefore, be based on detailed observation, reason, and experience [5]. Galen of Pergamum (129–216 B.C.E.) studied anatomy, surgery, drugs, and Hippocratic medicine in detail. His views influenced medical thinking for the next 1500 years. During the Renaissance, physicians increasingly relied on observation and experimentation to understand the human body and develop new techniques for diagnosis and treatment. The discovery of antibiotics, antiseptics, and other drugs in the 19th century accelerated the development of modern medicine, the latter being fueled further by advances in technology, research, a better understanding of the human body, and, most recently, the introduction of evidence-based medicine (EBM) [6].

EBM is one of the milestones that has shaped modern medicine. In this manuscript, following a summary of medical evolution and traditional concepts about EBM, we discuss how the foundational features of EBM have become crucial in current clinical practice and how they indulge in conjectural thoughts about the implementation of EBM in the current era of big data (large and diverse datasets huge in volume and rapidly growing over time) and precision medicine.

## 2. The EBM Concept

**Figure 1:**
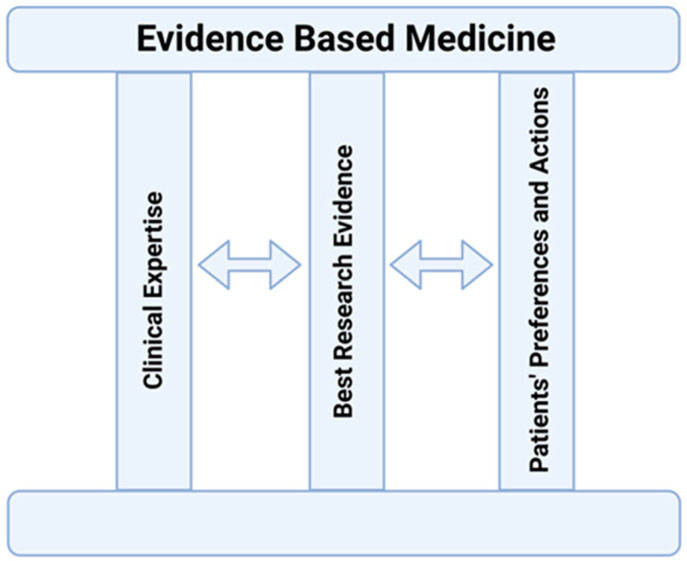
The interacting pylons of evidence-based medicine. Based on Ref [9].

In the first relevant article, it is stated that “Evidence-based medicine de-emphasizes (i) intuition, (ii) unsystematic clinical experience, and (iii) pathophysiologic rationale as sufficient grounds for clinical decision making, and stresses the examination of evidence from clinical research” [7]. Later, Sackett et al. emphasized the importance of clinical expertise, defined as the proficiency and judgment that individual clinicians acquire through clinical experience and clinical practice [8]. According to the latest model of EBM, clinical expertise must encompass and balance the patient’s clinical state, relevant research evidence, and the patient’s preferences and actions [9] (Figure 1).

Undoubtedly, EBM dramatically accelerated progress in several medical fields such as heart failure [10] and cancer [11]. However, the EBM concept is not without limitations [12] (Table 1) and has also raised important questions [13]. What is expertise? What is the ability to judge a patient’s clinical state? An agreed-upon interpretation of the terms used in this context is lacking. In addition, some evidence suggests that human decision-making is heavily influenced by unconscious mental processes, which occur without the intervention of our rational mind [10]. Although the latter unconscious processes may produce appropriate outcomes, they may also result in irrational choices.

## 3. EBM as Science

There are several definitions of science. Common to all definitions is the fact that science is a human mental activity aiming for truth or understanding [14], which implies a willingness or at least an attempt to either prove or, better still, disprove or falsify what one thinks or believes to know by using systematic observation, experimentation, and analysis of the natural world.

**Figure 2:**
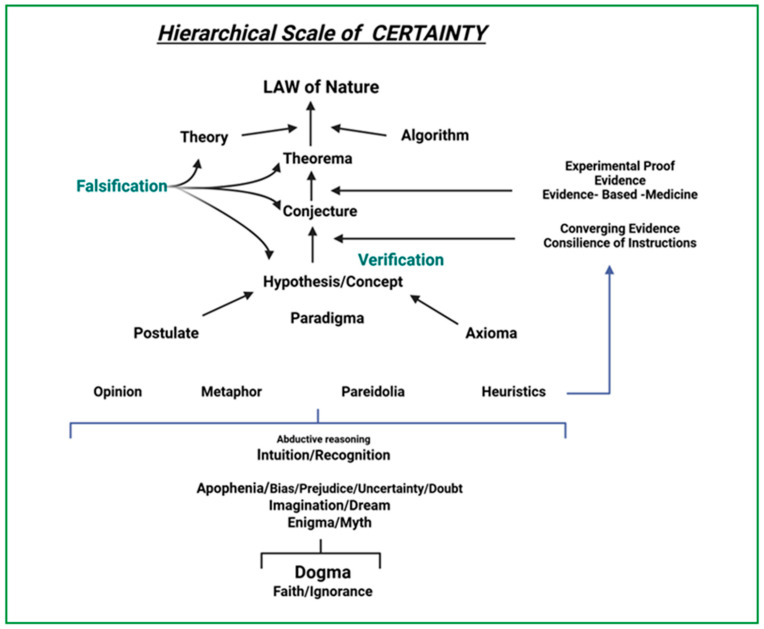
Exercises in scaling. Hierarchical levels from dogma to knowledge or ignorance versus certainty. To express different degrees of ‘certainty’ between the two extremes of dogma and law, various terms have been introduced in common usage. The terms are often used interchangeably, as subtle nuances in meaning may depend on whether the terms are used by a linguist, a scientist, a psychologist, a philosopher, a clergyman, or a commoner or pseudo-intellectual. See Addendum at the Supplementary Material for the explanation of terms in the figure

Science is based on strict inductive and deductive logical reasoning [15]. One starts with some premises, reaches a conclusion, and a theory is formed, which is subsequently tested and may be supported or refuted by the data. Undoubtedly, this is a simplistic description, as actual experiments never provide a precise result, necessitating the use of confidence levels to measure uncertainty in the estimate. In Figure 2, we have ordered some of the most frequently used terms in science (explained in Supplementary Materials Addendum 1) along a hierarchical scale. It illustrates our personal interpretation of how to best differentiate and rank the various terms depending on a hierarchical level of certainty between the two extremes of dogma–faith–ignorance and natural law. Note that in such a scaling exercise, one cannot entirely ignore some subjective interpretation. Hence, let us be clear about the lack of clarity.

***(i) The perplexing role of statistics.*** Misconceptions in medicine are partly due to the use of statistics as a fundamental research tool. Claude Bernard, in his book *An Introduction to the Study of Experimental Medicine*, stated: “Coincidences, it is said, can play such a large role in the causes of errors in statistics that one must only draw conclusions from large numbers. But the doctor has no use for what is called the law of large numbers, a law which, according to the expression of a great mathematician, is always true in general and false in particular. Which means that the law of large numbers never learns anything for a particular case” [16].

In a provocative paper titled ‘Why most published research findings are false’, John Ioannidis warns that “that there is increasing concern that most current published research findings are false, since the probability that a research claim is true may depend on study power and bias, the number of other studies on the same question, and, importantly, the ratio of true to no relationships among the relationships probed in each scientific field” [17].

***(ii) Limitations of randomized controlled trials***. Randomized controlled trials (RCTs) have been considered the gold standard of medical research [18]. Unfortunately, RCTs are limited by post-randomization bias, the risk of overlooking biases, and restricted generalizability, which is feasible only in simple systems, or when the conditions are exactly replicated [19]. Further, even well-designed RCTs often face technical limitations. Conducting an RCT timely generating evidence is not always feasible owing to several difficulties (e.g., cost, slow patient enrollment, ethical barriers, etc.), and by the time of completion and publication, many RCTs are obsolete and occasionally irrelevant to the current context [20].

***(iii) The role of meta-analysis***. Meta-analysis is currently considered a powerful tool for accumulating and summarizing knowledge in each research field. However, conclusions derived from meta-analyses are susceptible to the methodological quality of included studies, heterogeneity, publication bias, and the formulation of eligibility criteria [21]. Unfortunately, there are many published low-quality, underpowered, single-center trials, and this trend is deteriorating further [22]. In this regard, the Preferred Reporting Items for Systematic reviews and Meta-Analyses (PRISMA) statement published in 2020 included new reporting guidance that reflected advances in methods to optimize identification, selection, appraisal, and synthesis studies [23]. Furthermore, PRISMA modified the structure and presentation of the items to facilitate implementation [24].

As Douglas Altman recognized over 20 years ago, “much poor research arises because researchers feel compelled for career reasons to carry out research that they are ill equipped or trained to perform, but there is nobody to stop them” [25].

Despite these limitations and the incompleteness of knowledge, research findings remain a suggestive and potentially useful resource for health providers. As elegantly stated by Anaximander, considered one of the first scientists, the aforementioned limitations “do not imply that we cannot or must not trust our own thinking. On the contrary: our own thinking is the best tool we have for finding our way in this world. Recognizing its limitations does not imply that it is not something to rely upon. If instead we trust in ‘tradition’ more than in our own thinking, for instance, we are only relying on something even more primitive and uncertain than our own thinking. ‘Tradition’ is nothing else than the codified thinking of human beings who lived at times when ignorance was even greater than ours [26].”

## 4. Philosophy and EMB

Aristotle (384–322 BC), one of the greatest minds that ever existed, considered logic and philosophy to be the driving forces guiding medicine away from superstition and towards the scientific method [27].

Medicine and philosophy share their critical approach for asking questions, as both try to understand the mystery of our existence as human beings and of life in general using reason. However, human decision-making is heavily influenced by unconscious mental processes, which occur without the intervention of our rational mind. Furthermore, some major breakthroughs in science had been imagined as intuitive philosophical concepts long before there was any proof, disproof, or falsification. Copernicus, for example, used only his imagination and his logical point of view to develop his theory (the Heliocentric Solar System), which was viewed as most revolutionary. Only after a long time did Galileo Galilei prove it with his telescope and Newton describe the movements of the universe with his equations [28]. Yet, the philosophy of science has traditionally avoided granting an important role to imagination. Nevertheless, the attitude towards the role of imagination in science has changed in recent years. According to Arnon Levy and Godfrey-Smith, “imagination, our capacity to entertain thoughts and ideas ‘in the mind’s eye,’ is indispensable in science as elsewhere in human life. Indeed, common scientific practices such as modeling and idealization rely on the imagination to construct simplified, stylized scenarios essential for scientific understanding” [29].

The demarcation between science and philosophy, therefore, should be seen as a gray zone, in which some hypotheses or myths could not be proved initially, but were later either proved or disproved. Along with science teaching, classes in philosophy should therefore be an integral part of medical education.

Many contemporary scientists in biomedical sciences, guided by the availability of novel, powerful high-tech methodology, deviate from the traditional norm of first searching for proof or for falsification of novel ideas, hypotheses, or concepts. Instead, they prefer to focus first on blind data collection, relations, and statistical correlations, thereby postponing investigations of *how* and *why* until they have sampled all the facts. Hence the contemporary paradox and novel adagio in biosciences, “first wait to see what the data tell you”. This development is, no doubt, an unavoidable consequence of the ever more advanced methodological reductionism in science. Yet, any genuine progress in scientific research still necessitates a hypothesis, a philosophical disposition, and a lot of out-of-the-box thinking. As elegantly stated by John Floras in his 2021 Carl Ludwig Lecture, “preparation for and openness to serendipitous *n* = 1 observations can generate new and important insights into disease mechanisms and management [30]”.

## 5. Is EBM an Art?

It has been proposed that there are two intertwined stages in the formation of medicine: (i) accumulation of cases (empirical regularities) and (ii) setting the rules based on these cases (the normative stage) [31]. Therefore, a physician can return to the first stage if he finds no answers within the existent rules. In this unique situation, he can, within certain limits, use his *intuition*, a way of information processing involving implicit perceptual and cognitive processes. In clinical decision-making, it can be executed rapidly and automatically in the absence of a conscious mental will [32].

In line with general human decision-making models [33], intuition has been considered a major component of the creative process, but it has also been seen as something mysterious and possibly unknowable. Unfortunately, the intuitive art of medicine, as reflected in the therapeutic potential of the clinical encounter, has been marginalized. Healing through technological interventions has eclipsed healing through the clinician–patient relationship, i.e., one of the pylons of EBM [34].

The importance of the currently undermined clinician–patient relationship is supported by several lines of evidence, including the effectiveness of placebo. Novel treatments are validated by demonstrating their superiority over placebo controls. Interventions that fail this test are considered ineffective and equivalent to “no treatment”. Yet treatments that are no better than placebo controls may be superior to no-treatment (waiting list) interventions and even to standard medical or even surgical care. A typical example of the former is the effectiveness of placebo in improving patient-reported evaluations of tinnitus when using some standardized metrics [35]. By contrast, sham procedures related to rhinitis, chronic rhinosinusitis, recurrent acute rhinosinusitis, and nasal valve collapse are consistently associated with significant improvement in a variety of patient-reported outcome measures [36].

## 6. EBM in the ERA of Precision Medicine

Precision medicine is an evolving strategy incorporating individual genetic, environmental, and experiential variability for disease prevention and tailored treatment [37]. The implementation of precision medicine requires the construction of new tools for describing the health status of individuals and populations, based on the analysis of big data originating from ‘omics’, the exposome and social determinants of health, the microbiome, behavior and motivation, patient-generated data, as well as the array of data in electronic medical records.

***(i) Clinical trial design.*** Precision medicine necessitates significant changes in the design and interpretation of the findings of clinical trials. In the last decade, substantial progress has been achieved with the implementation of ‘master’ protocols, which provide a framework for coordinating the assessment of multiple treatments across various diseases or disease sub-types [38]. As these studies cut down on the time and resources needed to find and improve therapeutic candidates, they have become an essential component of contemporary drug research [39]. However, unraveling the molecular basis of disease requires a comprehensive understanding of small subsets of patients segregated by cellular processes and a fuller understanding of how these subsets relate to each other [40].

Regardless of the type of clinical trial, a serious problem that must be resolved is the fact that most clinical trials are conducted by the pharmaceutical industry. The release into the public domain of pharmaceutical industry documents, previously characterized as confidential, has given the medical community valuable information regarding the degree to which industry-sponsored clinical trials are misrepresented [41].

***(ii) Analysis and interpretation of trial data.*** Evidence in medicine originates from the analysis of research and of clinical trials, which generate data that may be structured or unstructured. The former has a predetermined schema, and it is extensive and freeform, and comes in diverse forms, whereas the latter, known as big data, does not fit into the typical data processing format. It provides a huge amount of datasets that cannot be stored, processed, or analyzed with traditional tools [42]. The generation of big data from several sources, such as medical imaging, “omics” and electronic medical records, renders the analysis of such data by man unfeasible and necessitates an increased reliance on machines. Due to the huge size and complexity of omics data and the dataset of patient features required for precision medicine, they cannot be analyzed directly by doctors. Artificial intelligence (AI), a computational program with the ability to process functions deemed typical of human intellectual functions [43], will be used to diagnose diseases, develop treatment plans, and assist clinicians with decision-making [44,45].

As the medical community increasingly relies on AI to aid with diagnosis, treatment, and research, it is fundamental for all medical professionals to have a better understanding of the statistical underpinnings of such platforms, including both non-generative AI models performing computations based on input data (e.g., image classification) and generative AI models producing “new” results (e.g., formerly unknown disease clusters) in order to interpret data.

***(iii) Communication of medical data.*** Accurate communication of data, even from appropriately designed studies, remains problematic, however. Language is not a monolithic tool for communication, as it is not always interpreted by everybody in the same way. Words derive their meaning from how they are being used in daily life [46]. As a result, one is never able to obtain absolute truth by using language. The above inherent limitations in human communication, aggravated by conflicts of interest among professional committee members, may affect the implementation of EBM guidelines, despite international standards related to evidence evaluation, transparency, and bias reduction [47].

Citing the philosopher Karl Popper, “Although clarity is valuable in itself, exactness or precision is not…Linguistic precision is a phantom, and problems connected with the meaning or definition of words are unimportant [48].” In contrast to the philosopher Popper, we as scientists feel that in this linguistic labyrinth, one should nevertheless try to create some order out of chaos.

***(iv) Large language models (LLMs) and digital twins***. Chatbots are programmed to understand and respond to user queries, making them super handy for answering frequently asked questions. LLMs, like GPT-4 (Generative Pretrained Transformer 4), are the geniuses of the AI world, as they represent a discipline of machine learning that comprehends linguistic patterns, semantics, and contextual meaning through processing of tremendous amounts of data in the form of text [49]. GPT-4 may transform healthcare by providing unprecedented routes to synthesize and distribute medical knowledge. To use a chatbot, one (usually a human) starts a “session” by introducing a query usually referred to as a “prompt” in natural language, and subsequently the chatbot gives a natural-language “response,” normally within 1 s, that is relevant to the prompt [50]. The session comprises an exchange of prompts and responses reminiscent of a conversation between two individuals. These AI-powered systems can refine clinical workflows, assist in reaching clinical decisions, and eventually improve patient outcomes. The findings of studies highlight the usefulness of LLMs in clinical decision-making by providing valuable comprehension, which enables healthcare providers to reach more informed treatment decisions [51,52]. In a study including 92 practicing physicians randomized to use either GPT-4 plus conventional resources or conventional resources alone to answer five expert-developed clinical vignettes, those using the LLM scored significantly higher compared to those using conventional resources [53]. In addition, chatbots hold promise in modifying health behaviors to promote mental health. In a randomized controlled trial including adults with major depressive disorder, generalized anxiety disorder, or clinically high risk for feeding and eating disorders, participants were randomly assigned to a 4-week chatbot (Therabot) intervention (*n* = 106) or waitlist control (*n* = 104). Therabot was well utilized (average use > 6 h), and the participants rated the therapeutic intervention as equivalent to that of human therapists [54].

LLMs also demonstrate significant potential in patient education and engagement by creating accessible educational materials, interpreting complex medical information and enhancing communication between patients and healthcare providers [55]. Key considerations include enhancement of accuracy, adoption of robust evaluation metrics beyond readability, and the integration of LLMs with clinical decision support systems to improve real-time patient education [56].

**Figure 3:**
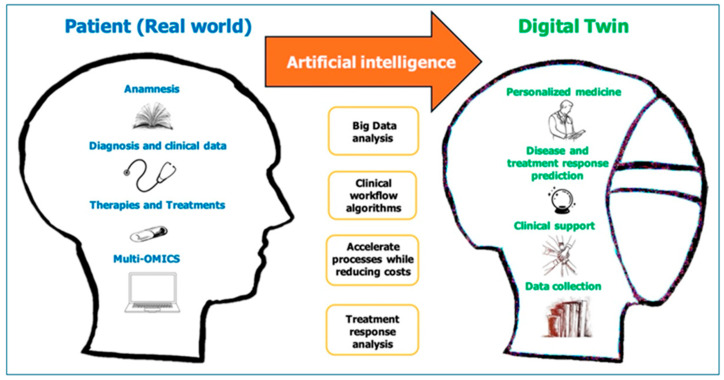
Envisioned digital twin for healthcare (DT4H). A DT4H is a virtual representation of an individual that renders feasible early intervention and prevention, monitoring and prediction of a health trajectory, and dynamic modeling of a possible treatment modality. Adapted from Ref. [59].

However, despite the undoubtable LLM capabilities, concerns have been expressed about their use in healthcare. GPT-4 suffers from hallucinations, namely, the fabrication of references and justifications for its rationale [57]. These flaws are most apparent when GPT-4 malfunctions in simple mathematical computations and logic statements and, at the same time, presents the results in a persuasive manner [58]. While current efforts are focused on additional training to alleviate these errors, it might only shift these hallucinations to more complex scenarios in which they are more likely to pass unnoticed, resulting in potentially more severe consequences. An application in engineering, the digital twin, has been proposed as a potential solution in medicine—the so-called medical digital twin (MDT) (Figure 3) [59,60]. MDTs, by combining diverse health data streams and disease modeling, generate a patient copy, which augments clinical decision-making, leads to precision treatment, and at the same time attenuates the workload of healthcare providers. MDTs, by generating a detailed and precise disease model (“the patient-in-silico”) can be used to simulate disease severity and progression as well as the different treatment outcomes at the patient level [61].

## 7. Conclusions and Future Perspectives

Medicine has evolved from ancient healing practices rooted in superstition and religious beliefs to the current sophisticated, science-based discipline. This is due to the advancements in various fields like biology, chemistry, and technology, which have led to a deeper understanding of disease, more effective treatments, and a greater emphasis on prevention. However, the rapid expansion of medical knowledge, together with the increasing patient complexity (advanced age, several coexisting morbidities, polypharmacy, etc.), renders each patient a “big data” challenge, with vast amounts of information on past and current states [62].

In the era of big data challenging the limits of the human mind, major EBM principles must be based on (a) the systematic identification, analysis, and utility of big data using AI; (b) the magnifying effect of medical interventions by means of the physician–patient interaction, the latter being guided by the physician’s expertise, intuition, and philosophical beliefs; and (c) the patient preferences, since, in healthcare under precision medicine, the patient will be a central stakeholder contributing data and actively participating in shared decision-making.

Machine use will be unavoidable during this process. As for the physician, the reasons are obvious and previously analyzed. As for the patient, it is highly likely that with the widespread use of AI applications, such as LLMs, patients may consult these machines about their illness. Although patient education contributes to treatment, the risk of misunderstanding the advice obtained from the machine, or even receiving wrong and occasionally dangerous information, is not negligible.

However, despite the undoubtable recognition of LLM capabilities, concerns have been expressed about their use in healthcare [63]. During the process of mitigating these risks, some unresolved issues are related to “human values” embedded in AI models and how the “LLM values” may not line up with “human values”, even if LLMs no longer confabulate and the toxic output has been eliminated [64].

**Figure 4:**
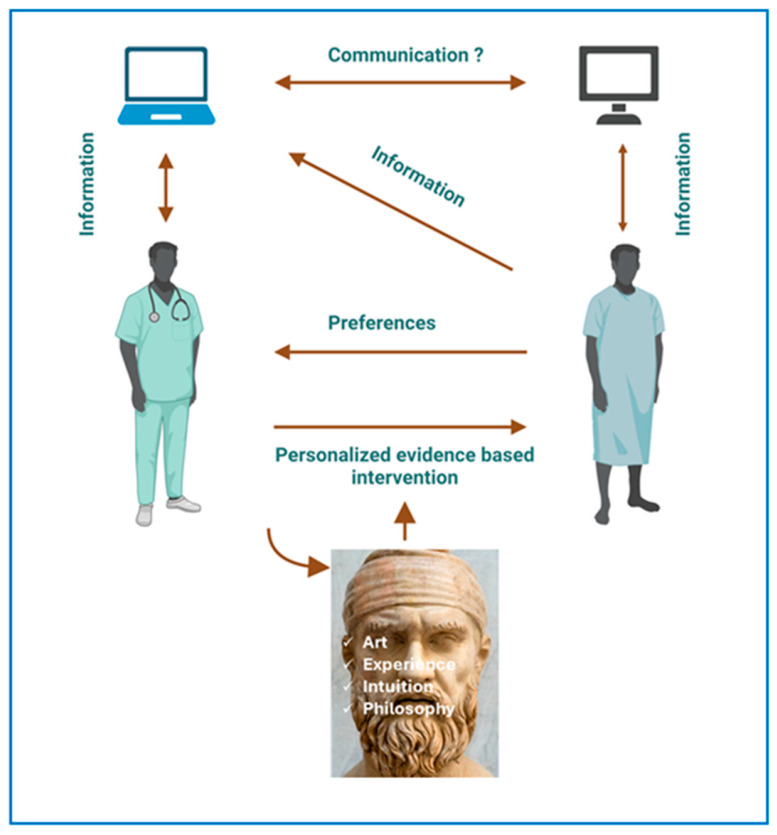
Evidence-based medicine in the era of precision medicine. Machines will be used by the physician and most likely the patient. Medical intervention should be evidence-based and also consider patient preferences. The Hippocratic art of medicine driven by physician experience, intuition, and philosophical reasoning will be fundamental in protecting human values during physician–patient interaction. Hippocrates’ photo taken by Marian Florinel Condruz and obtained from the Unsplash website.

If the aim of EBM is to prevent disease, relieve suffering, care for the ill, and avoid premature death regardless of cultural, political, and economic circumstances, it needs more than science and technology (Figure 4). As Hippocrates stated, “Wherever the art of medicine is loved, there is also a love of humanity” [65]. The art of physician–patient communication can dramatically influence patient wellbeing, i.e., through the time spent with the patient, verbal and nonverbal interpersonal communication, and the genuine understanding of the patient’s demands [66].

## Figures and Tables

**Table 1:** Limitations of evidence-based medicine.

Many bedside decisions reached by clinicians are complex, driven by combinations of diagnostic evaluations and therapeutic interventions
Evidence is not readily available to support bedside decision-making
Broad literature aggregated within reviews often does not extrapolate well to an individual clinical situation
When rapid decision-making is required, the clinician is required to make ad hoc decisions without consulting the available evidence
If the observed effect is real and the original *p*-value is <0.05, attempts to replicate the finding will produce a not statistically significant *p*-value in approximately half of the time
Clinical trials often require enormous effort over a protracted period and may be outdated when they finally become publicly available

Table compiled based on data from ref. [12].

## Supplementary Materials

The following supporting information can be downloaded at https://www.mdpi.com/article/10.3390/jcm14145094/s1, File S1: Addendum 1.
