# Supplementary material for: Evidence-Based Medicine: Past, Present, Future
Source: J Clin Med. 2025 Jul 17;14(14):5094. doi: 10.3390/jcm14145094 (PMC12295464; doi:10.3390/jcm14145094)
Supplement: Supplementary file 1 [file jcm-14-05094-s001.zip › jcm-3736622-supplementary.pdf]

ADDENDUM to Figure 2. Summary of the various definitions and/or interpretations of the terms depicted in Figure 2.

-**Law, natural law**: Description of phenomena occurring in nature and proven by scientific method (e. g. gravity, natural selection; hence, not the momentary, fleeting human/judicial laws).

-**Algorithm:** Rule for processing input data to optimize output.

-**Theory:** A scientifically acceptable principle or group of principles offered to explain phenomena. Any scientific theory necessitates (experimental or other) proof. A new theory starts from trying to solve problems (Popper). Hence first tell what the problem is.

-**Theorem:** A statement in mathematics that has been proven on the basis of previously established statements, such as other theorems, or of generally accepted statements, such as axioms. As theorems are required to be proved, a theorem is fundamentally deductive, in contrast to a scientific law, which is experimental.

-**Evidence based medicine:** Application of the best available research to clinical care, which requires the integration of evidence with clinical expertise and patient values.

-Conjecture: Unproven idea (i.p. in mathematics) from Latin 'cum' and 'iacere' , i.e. throw together (guess, divine, assume). Some conjectures can hardly, if ever, be proven through experiments. In conceiving a conjecture, many scientists describe their approach as stochastic, i.e. find the truth by transgressing different scientific disciplines and applying statistics to apparently related, educated guesses, and by integrating all of it into one single conjecture

-Converging evidence: Preponderance of evidence emerging from numerous converging lines of inquiry pointing to the same conclusion; or, a process of independent lines of inquiry converging to a single conclusion. ["Consilience of Inductions", by William Whewell, XIXth-century Philosopher of Science]. Consilience, i.e. a coincidence, or, the act of concurring [Latin: cum; salire, to leap; to jump over]. Induction, i.e. process of reasoning or drawing a conclusion from particular facts or individual cases.

-Consilience of inductions: Occurs when an induction (the process or action of bringing about or giving rise to something), obtained from one class of facts, coincides with an Induction obtained from another different class.

-Paradigma: A model, a general frame for developing a theory; any paradigm can be replaced by better ones.

-Axioma: Unproven, indemonstrable intuitive truth, accepted without proof as fundamental principle (e.g., two parallel lines don't cross.) Most breakthrough theories in physics were first introduced as never questioned axiomata but continue to be subjected to either strict proof or falsification.

-Aphorism: A short, pithy and pointed sentence containing some important truth or precept; a definition; a short, concise statement of a principle.

-Postulate: Suggestion or acceptance that a theory or idea is true as a starting point for reasoning or discussion. Indemonstrable, but necessary to understand some reasonings.

-Opinion: A thought or belief about something in search for the truth; to be distinguished from knowledge of the truth.

-Metaphor, from the Greek μεταφορά, "transfer", from μεταφέρω, "to carry over", "to transfer"; from μετά, "after, with, across + φέρω, "to bear", "to carry". Metaphor is a poetically or rhetorically ambitious use of words, a figurative as opposed to literal use. It has attracted more philosophical interest and provoked more philosophical controversy than any of the other traditionally recognized figures of speech (Stanford Encyclopedia of Philosophy, 2017). A metaphor is a figure of speech that refers, for rhetorical effect, to one thing by mentioning another thing. It may provide clarity or may identify hidden similarities between two ideas. Where a simile compares two items, a metaphor directly equates them, and does not use "like" or "as" as does a simile. In 'De Poetica' (458-459) Aristotle wrote…"But the greatest thing by far is to be a master of metaphor. It is the one thing that cannot be learnt from others; and it is also a sign of genius, since a good metaphor implies an intuitive perception of the similarity in dissimilars". Aristotle warns, however, that metaphors should be used with moderation, and, if used improperly,

may provoke laughter. Allegory, antithesis, catachresis, hyperbole, and parable are special forms of metaphor. Proper use of metaphors is essential in scientific research.

-Pareidolia: A tendency to perceive a specific, often meaningful image in a random or ambiguous visual pattern (e.g. the Rorschach psychological ink-test).

-Apophenia: The human tendency to perceive connections and meaningful, but non-existing patterns between unrelated things. Confirmation bias and uncertainty are forms of apophenia, e.g. uncertainty is when a gambler wonders what his chances are to winning at the roulette table. The term (German: Apophänie) was coined by psychiatrist Klaus Conrad in his 1958 publication on the beginning stages of schizophrenia. He defined it as "unmotivated seeing of connections accompanied by a specific feeling of abnormal meaningfulness". He described the early stages of delusional thought as self-referential, over-interpretations of actual sensory perceptions, as opposed to hallucinations. Apophenia has come to imply a universal human tendency to seek patterns in random information, such as gambling. It is our nature as human beings to look for connections—and often to discern them where none actually exist, something known as illusory correlation. The ability to discern a true pattern quickly can be a time saver—and one would expect evolutionarily that it may have been a life saver as well. (Wikipedia).

-Heuristics: Mental shortcuts which allow individuals to make fast decisions but may also lead to cognitive biases (e.g., heuristic reasoning, heuristic techniques). Heuristic assumptions may serve as a strategy to improve problem-solving; it may use trial-and-error and feedback

techniques. Contrary to algorithms (which always work), heuristics are specific problem-solving strategies applicable in specific situations where a solution cannot necessarily be guaranteed but where heuristic reasoning may provide means for the most feasible solution.

-Abductive reasoning: Reasoning toward the most plausible hypothesis; is like in chess game, when it has already started, and the problem-solver is trying to figure out what has happened; one has to reason backwards to imagine these possibilities; it helps creating hypotheses.

-Recognition: Identification of someone or something from previous encounters or knowledge. Humans recognize objects because they have seen similar objects in the past, stored in long-term memory.

-Intuition: The human capacity for direct knowledge, for immediate insight without observation or reason…Captain Kirk Principle: "Intellect is driven by intuition; intuition is directed by intellect. Intuition is the key to knowing without knowing how you know". Without intellect, our intuition may drive us unchecked into emotional chaos; without intuition, we risk failing to resolve complex dynamics and dilemmas. Hence, intellect and intuition, i.p. 'creative' intuition, are complementary, not competitive." [The Captain Kirk Principle, by Michael Shermer, in: Scientific American, Dec 2002, p.20.]

-Imagination: A powerful, typical human characteristic, in which -according to Hegel- the most inner fuses with the exterior. "Toutes les grandes découvertes ont d'abord été rêvées" (Gaston Bachelard). Too much imagination can, however, turn into illusion, i.e. to cite Pascal: "cette partie décevante dans l'homme, cette maîtresse d'erreur et de fausseté…" (L.Vander Kerken , S.J. in DS-Letteren)

-Dream: Fantasy about something greatly desired. With August Kekulé (1858):" let us learn to dream; but let us beware of publishing our dreams till they have been tested by the waking understanding."

-Enigma, riddle: Something mysterious or difficult to understand. To borrow W. Churchill's famous observation on Russia, 'a riddle wrapped in a mystery inside an enigma" …Elusive?

-Myth: An imaginary tale, organized and coherent along a psycho-affective logic, and pretending (claiming) to be based on reality and truth.
